# An Overview of Adipose Tissue ACE2 Modulation by Diet and Obesity. Potential Implications in COVID-19 Infection and Severity

**DOI:** 10.3390/ijms22157975

**Published:** 2021-07-26

**Authors:** Saioa Gómez-Zorita, Iñaki Milton-Laskibar, Laura García-Arellano, Marcela González, María P. Portillo

**Affiliations:** 1 Nutrition and Obesity Group, Lucio Lascaray Research Center, Department of Nutrition and Food Science, Faculty of Pharmacy, University of the Basque Country (UPV/EHU), 01006 Vitoria-Gasteiz, Spain; laurai.arellano.garcia@gmail.com (L.G.-A.); mariapuy.portillo@ehu.eus (M.P.P.); 2 CIBERobn Physiopathology of Obesity and Nutrition, Institute of Health Carlos III (ISCIII), 28029 Madrid, Spain; 3 BIOARABA Health Research Institute, 01006 Vitoria-Gasteiz, Spain; 4 Precision Nutrition and Cardiometabolic Health, IMDEA-Food Institute (Madrid Institute for Advanced Studies), Campus of International Excellence (CEI) UAM+CSIC, Spanish National Research Council, 28049 Madrid, Spain; 5 Nutrition and Food Science Department, Faculty of Biochemistry and Biological Sciences, National University of Litoral and National Scientific and Technical Research Council (CONICET), Santa Fe 3000, Argentina; maidagon@fbcb.unl.edu.ar

**Keywords:** SARS-CoV-2 infection, COVID-19, adipose tissue, obesity, diet composition, caloric restriction, ACE2, renin–angiotensin system

## Abstract

The present review is aimed at analysing the current evidence concerning the potential modulation of obesity and/or diet in adipose tissue ACE2. Additionally, the potential implications of these effects on COVID-19 are also addressed. The results published show that diet and obesity are two factors that effectively influence the expression of *Ace2* gene in adipose tissue. However, the shifts in this gene do not always occur in the same direction, nor with the same intensity. Additionally, there is no consensus regarding the implications of increased adipose tissue ACE2 expression in health. Thus, while in some studies a protective role is attributed to ACE2 overexpression, other studies suggest otherwise. Similarly, there is much debate regarding the role played by ACE2 in COVID-19 in terms of degree of infection and disease outcomes. The greater risk of infection that may hypothetically derive from enhanced ACE2 expression is not clear since the functionality of the enzyme seems to be as important as the abundance. Thus, the greater abundance of ACE2 in adipose tissue of obese subjects may be counterbalanced by its lower activation. In addition, a protective role of ACE2 overexpression has also been suggested, associated with the increase in anti-inflammatory factors that it may produce.

## 1. Introduction

The COVID-19 pandemic that started in December 2019 in Wuhan (Hubei province, China) has caused a global health-crisis, resulting in more than 160 million confirmed cases and causing almost 3.5 million deaths worldwide by May 2021 [1]. The disease is produced by the severe acute respiratory syndrome coronavirus 2 (SARS-CoV-2), an enveloped, positive-sense, single-stranded RNA virus sharing 50% and 79% of its genome sequence with the Middle East respiratory syndrome coronavirus (MERS-CoV) and the SARS-CoV, respectively [2]. It is estimated that 80% of the cases are asymptomatic or develop mild symptoms, while 10–20% of the cases develop severe symptoms (including pneumonia) and a further 5% of the cases develop acute respiratory syndrome distress, multi organ failure or septic shock [3,4]. As far as SARS-CoV-2 transmission is concerned, aerosols have been identified to play a major role in human-to-human transmission [5].

**Figure 1:**
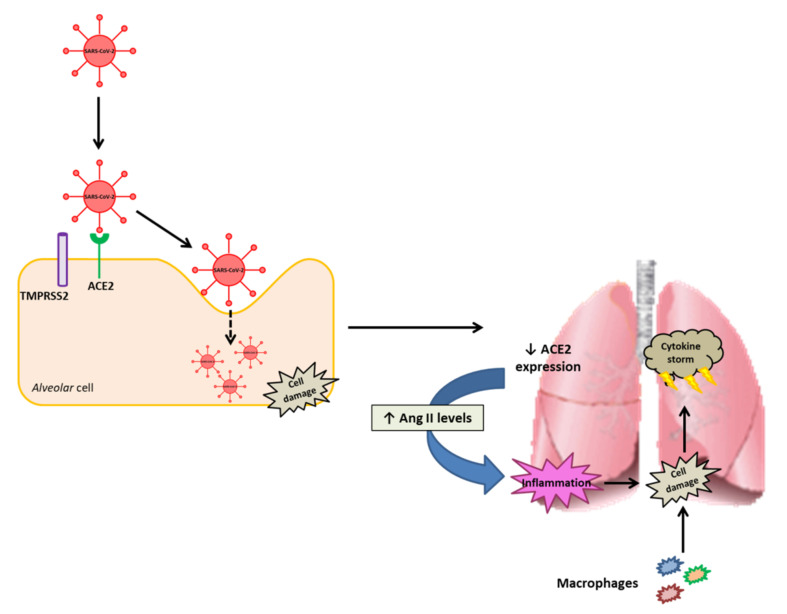
Summarised schematic representation of the SARS-CoV-2 viral infection process and derived outcomes in pulmonary tissue. ACE2: angiotensin-converting enzyme 2, Ang II: angiotensin II, TMPRSS2: transmembrane serine protease.

Angiotensin-converting enzyme 2 (ACE2) has been identified as a major player in human SARS-CoV-2 infection (Figure 1). In this regard, the viral spike protein of SARS-CoV-2 interacts with ACE2 extracellular domains, facilitating the entry of the virus into alveolar cells [6], a process in which the transmembrane serine protease 2 (TMPRSS2) protein is also involved [7]. Indeed, the higher binding affinity of SARS-CoV-2 towards ACE2 is considered among the factors leading to the rapid spread of the disease worldwide [8]. Once the virus enters into the cell, the expression of ACE2 is dramatically decreased in pulmonary tissue, resulting in higher circulating angiotensin II levels that in turn can aggravate lung tissue inflammation [9]. Moreover, the cell damage derived from this enhanced pulmonary tissue inflammation induces macrophage recruitment, which is known to trigger the so-called “cytokine storm”, commonly resulting in further cell-deterioration and inflammation [10].

**Figure 2:**
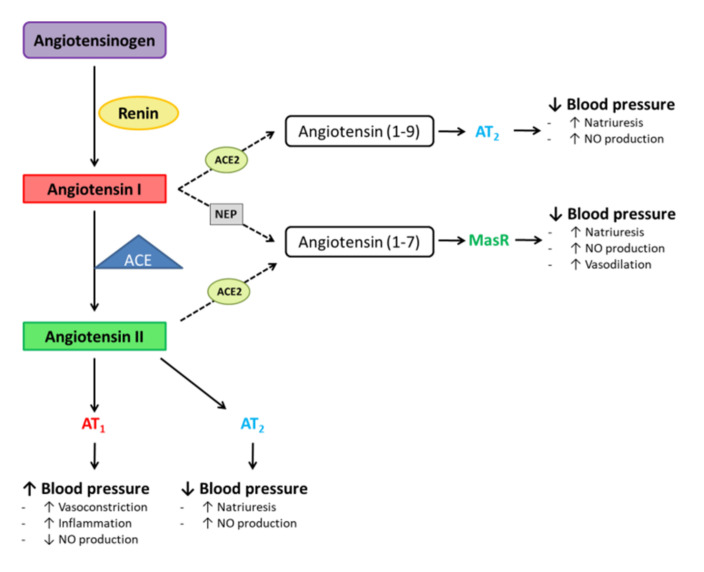
Schematic representation of the renin–angiotensin system. ACE: angiotensin-converting enzyme, ACE2: angiotensin-converting enzyme 2, AT_1_: angiotensin receptor 1, AT_2_: angiotensin receptor 2, MasR: Mas receptor, NEP: neprilysin, NO: nitric oxide.

ACE2 is part of the renin–angiotensin system (RAS), which in turn is a major regulator of electrolyte balance and blood pressure [11]. Under physiological conditions, the angiotensinogen (AGT) produced in the liver undergoes renin-mediated cleavage, resulting in angiotensin I. Then, the angiotensin-converting enzyme (ACE), which is located in the pulmonary vasculature endothelium, converts angiotensin I in angiotensin II (Figure 2). Subsequently, angiotensin II binds to two angiotensin receptors (AT), angiotensin receptor 1 (AT_1_) being the receptor mediating the majority of the angiotensin II physiological effects, such as vasoconstriction, growth stimulation or water and salt retention. These receptors are widely distributed in various organs and tissues, such as heart, liver, lungs or blood vessels. By contrast, angiotensin receptor 2 (AT_2_) exerts vasodilatory effects and inhibits cell growth and differentiation once having bound angiotensin II, somehow counterbalancing the effects of AT_1_ [11]. In addition, angiotensin I and II have the ability to produce angiotensin 1-9 and angiotensin 1-7. In this regard, angiotensin I is cleaved by ACE2 to produce angiotensin 1-9, which binds AT_2_, triggering blood pressure reduction through increased natriuresis and nitric oxide (NO) production. With regard to angiotensin II, it can be cleaved by ACE2 to produce angiotensin 1-7, which binds Mas receptor (MasR) and reduces blood pressure. Interestingly, angiotensin 1-7 can also be produced from angiotensin I, a process that is mediated by neprylsin (NEP) [12].

The ACE/Ang-II/AT1 receptor axis and AT2 receptor are known as the “harmful” or classical arm of the RAS and the “protective” arm of the RAS, respectively. Up-regulation of Ang II/AT1R-mediated signalling has been identified in pulmonary diseases [13]. Indeed, the activation of the ACE/Ang II/AT1R cascade is associated with increased risk of pneumonia and worse prognosis [14]. After SARS-CoV-2 infection, an imbalance in both arms of the RAS system takes place. Thus, COVID-19 shares this pathophysiological pathway with pulmonary hypertension and pulmonary fibrosis, both commonly seen in chronic lung diseases such as chronic obstructive lung disease, and with acute lung diseases, especially acute respiratory distress syndrome [15]. In the matter of the damage produced by COVID-19, data obtained in several studies have demonstrated that once a subject is infected, an uncontrolled systemic inflammatory response takes place, which is triggered by the immune system [13]. This inflammatory response mainly affects the lungs, especially harming the alveolar epithelium and pulmonary interstitial arteriolar walls, which results in reduced lung capacity and function impairment [13]. Regarding inflammation, it has been demonstrated that ACE2 attenuates this process, protecting against lung injury of acute respiratory distress syndrome and restores the balance in RAS [15]. Thus, appropriate ACE2 activity might be decisive in preventing immune-induced damage and ensuring tissue repair [16]. Taking all that into account, the scientific community is testing ACE inhibitors, ACE-2 agonists and Mas or AT2 receptor activators, among others [17,18], in hospitalised patients. It would also be of interest to study the potential therapeutic possibilities of ACE2 metabolites, including angiotensin (1-7), des-Arg (9)-bradykinin, apelin and dynorphin [15].

COVID-19 derived impairments are not limited to pulmonary tissue, and this is mainly due to the specific characteristics of SARS-CoV-2 spike protein, which enables the virus to provoke multi-organ failure [19,20]. It is noteworthy that different studies have demonstrated the tendency amongst obese subjects to develop a more severe COVID-19 infection, the body mass index (BMI) of a subject being related to the risk of admission in intensive care units (ICU) or even death [21]. In this regard, it has been suggested that the acute inflammation triggered by COVID-19 is amplified in obese subjects due to the subjacent chronic low-grade inflammation that is commonly associated to this disease, leading to a more severe disease phenotype [22]. It has been proposed that this effect is driven by the pro-inflammatory cytokine dysregulation underlying the obesity-derived low-grade inflammation, which may overreact to SARS-CoV-2 infection [22,23]. The aforementioned effects of obesity on COVID-19 seem to be independent from further health alterations related to this disease, such as diabetes or cardiovascular diseases [24], which in turn highlights the connection between excessive adipose tissue accumulation and COVID-19 development [25].

Besides the apparent higher risk of obese subjects to develop a more severe COVID-19 infection, it has been suggested that obesity may also lead to a higher risk of SARS-CoV-2 infection. In this regard, it must be noted that in addition to the lungs, ACE2 is also expressed in white adipose tissue, where it shows a higher expression in comparison to that found in pulmonary tissue [26]. Moreover, studies conducted in animal models (rodents) have demonstrated that the adipose tissue ACE2 can be up-regulated in diet-induced obesity (high-fat feeding) [27], which converts adipose tissue in a potential target and a reservoir for COVID-19 [23].

At this point, it would be plausible to assume that obese subjects may be more prone to get infected by SARS-CoV-2, and to develop a more severe COVID-19 infection. However, the relationship between both diseases has become more complex. Even though obese subjects are more likely to develop COVID-19 pneumonia, their mortality rate has been reported to be lower than that of non-obese subjects [23]. Similar observations were also described regarding ICU admissions, where obesity was related to the frequency of admissions, but not with hospital stay length or mortality rate [28]. Additionally, there is no consensus to date in regard to whether higher ACE2 may result in a negative (higher amount of viral infection targets) or a positive (regulation of RAS system) effect in relation to the SARS-CoV-2 infection [29,30].

In this scenario, the aim of the present review article is to analyse the current evidence with reference to the potential modulation that obesity and/or diet may exert in adipose tissue ACE2 expression. Additionally, the potential implications of ACE2 expression and modulation in COVID-19 are also addressed.

## 2. Influence of Different Dietary Patterns and Weight-Loss Strategies in Adipose Tissue ACE2 Levels. Evidence from Preclinical Studies

A number of studies have addressed the relationship between different feeding patterns in adipose tissue renin–angiotensin system (RAS) components, due to the suggested link between the latter and metabolism regulation, as well as the development of inflammatory and cardiovascular diseases [31,32].

### 2.1. Preclinical Studies Carried Out in Dietary Rodent Models

#### 2.1.1. Dietary Composition

Several preclinical studies have been carried out to analyse the effects of dietary composition on RAS components (Table 1). One such study was conducted by Gupte et al. [27], in which the authors aimed at investigating whether ACE2 expression in adipose tissue could be regulated by high-fat diet feeding. For this purpose, male C57BL/6 mice were fed with a low-fat diet (LFD) or a high-fat diet (HFD) (10 and 60% of energy as fat, respectively) for one week or four months. The results revealed that after four months of HFD feeding, the circulating levels of angiotensin peptides I, II and IV were significantly boosted. By contrast, this effect was not reported after a week of feeding. Moreover, the authors observed that HFD feeding not only induced a significant increase in body and epididymal adipose tissue weight, but also enhanced *Agt* mRNA levels in this adipose depot. Indeed, a significant build-up in this marker was also observed in mice fed with either the HFD for a week or for four months. In addition, the authors also found that, compared to their LFD-fed counterparts, *Ace2* mRNA levels were significantly increased in epididymal and subcutaneous adipose tissues of mice fed with the HFD for a week or for four months. With regards to the ACE2 protein expression, it was significantly increased in epididymal adipose tissue of mice fed with the HFD compared to the LFD-fed animals after a week, while the opposite was observed (a significant decrease in HFD-fed mice) after four months. Remarkably, ACE2 activity was also augmented in these same adipose depots after a week of HFD diet feeding, whereas no such differences were appreciated in none of the studied adipose depots after maintaining the animals for four months on this same diet. Finally, the authors also studied desintegrin and metalloproteinase domain 17 (Adam17) mRNA levels in epididymal adipose tissue, which is known to cleavage ACE2 from membranes. Results revealed that after four months of HFD feeding, the gene expression of *Adam17* was significantly increased in comparison to animals fed with the LFD.

The authors concluded that both short- and long-term HFD feeding influence adipose tissue ACE2 expression, although differently. Thus, while under a short-term HFD feeding, adipose tissue ACE2 gene and protein expressions as well as enzymatic activity were increased. In addition, under long-term HFD feeding, the upsurge in ACE2 gene expression was not accompanied by neither greater protein expression nor enzymatic activity. On the contrary, ACE2 protein expression was decreased.

The same group [33] further hypothesized that differential tissue-specific ACE2 regulation and shift in angiotensin II/(1-7) balance may differently exert an influence over obesity-related hypertension development in male and female mice. To prove such a hypothesis, male and female C57BL/6 mice were fed with a LFD or a HFD (10 or 60% of energy as fat, respectively) for four months. Once the experimental period was completed, the authors found significant body weight and fat mass growth in mice of both sexes that had been fed with the HFD, results that differed from those found in their LFD-fed counterparts. When analysing the plasma angiotensin II levels, the authors did not find sex differences between animals fed with the same diets. By contrast, and compared with their LFD-fed counterparts, a significant increase in this parameter was found in males fed with the HFD. Concerning plasma angiotensin (1-7) levels, and compared to males receiving the same diet, a significant reduction was found in this parameter in females fed with the LFD, while no differences were found between HFD-fed males and females. Interestingly, significantly lower plasma angiotensin (1-7) levels were found in males fed the HFD when compared to the LFD-fed males, whereas the opposite was appreciated in females. Finally, no changes in adipose tissue ACE2 activity were reported amongst the male mice. Contrarily, the females fed with the HFD showed a greater activation than those in the LFD. Moreover, the LFD-fed female mice showed a significant reduction in ACE2 activity when compared to the males fed with the same diet.

Based on the results obtained, the authors concluded that a sexual dimorphism exists in the diet-induced obesity-mediated angiotensin II/(1-7) balance. Thus, in males, the HFD feeding shifts the balance towards Ang II/AT1R stimulation and plasma angiotensin (1-7) suppression, whereas in females, the same dietary pattern enhances plasma angiotensin (1-7) levels.

In the study published by Pinho et al. [34], male FVB/N mice were fed: a diet that followed the macronutrient recommendations for mature rodents (AIN-93M); a standard with a lower carbohydrate content than the AIN-93 M diet; a high-glucose diet; a high-protein diet; or a high-lipid diet for two months. At the end of the experimental period, a lower body weight was found in the animals fed with the high-glucose diet compared to those receiving the AIN-93M, the high-protein or the high-lipid diets. Retroperitoneal adipose tissue weight remained unchanged, whereas compared to the group fed the standard diet, increased epididymal adipose tissue weight was found in the groups fed with the AIN-93M and high-protein diets, although not in those fed with the high-glucose or the high-lipid diets. Moreover, reduced blood triglyceride levels were also observed in the high-lipid diet-fed group when compared to the AIN-93M, high-protein and high-glucose-diet fed animals. The authors also studied the gene expression of different RAS components in epididymal adipose tissue. In this regard, the *Ace* mRNA levels found in the group fed with the high-lipid diet was significantly higher than that found in the groups fed with the standard or AIN-93M diets. By contrast, a significant reduction in *Ace2* gene expression was reported in these animals. No differences among the other groups were found. This result clearly differs from that obtained by Gupte et al. (2008) whose mice were fed with a HFD for four months. No changes were observed concerning renin and *Agt* gene expression among the experimental groups.

Based on what the results yielded, the authors concluded that high-lipid feeding modulates the expression of RAS components in adipose tissue, and it may therefore participate in obesity-related health alterations. In addition, the reduction in ACE2 produced by this dietary pattern would reduce the benefits associated with this enzyme, which is known to counterbalance the RAS action.

Other studies have been conducted using rats of different strains (instead of mice) as experimental models. Among them, Zhang et al. [35] carried out a study with male Sprague–Dawley rats fed with a standard chow diet or a HFD (mainly fat from lard) for six months. At the end of the experimental period, as expected, the body weight was greater in rats fed with the HFD. Surprisingly, the weight of the adipose tissue was not determined. Serum glucose, insulin, total cholesterol, LDL-cholesterol, HDL-cholesterol, triglyceride and free fatty acid levels were also higher in rats fed with the HFD. In epididymal adipose tissue, ACE2 gene and protein expression were augmented in animals fed with the deleterious diet.

In the experiment published by Coelho et al. [36] male Wistar rats were fed with a standard chow diet and received tap water or water containing 20% sucrose solution for a month in order to produce a model of metabolic syndrome with high plasma angiotensin II levels. At the end of the experimental period, the authors found enhanced plasma levels of insulin, triglycerides, leptin, angiotensin I and angiotensin II, as well as increased plasma renin and ACE enzyme activities. Moreover, epididymal adipose tissue weight was significantly increased in the sucrose-fed animals, as it was the de novo fatty acid synthesis. A significant boost in angiotensin I, II and (1-7) levels in the epididymal adipose tissue was also observed. By contrast, no differences in *Ace* mRNA levels were found. ACE protein expression and activity were higher in rats receiving sucrose, although the protein expression and the activity of ACE2 were decreased. *Agt* gene expression was also decreased in the sucrose fed animals. In conclusion, even though *At1* and *At2* gene expression remained unchanged, their protein expression was significantly increased in the sucrose fed animals. Based on these results, the authors determined that sucrose intake not only augmented epididymal adipose tissue weight and de novo fatty acid synthesis in rats, but also regulated the ACE/ACE2 balance towards a vasodilator/antiproliferative effect.

**Table 1:** Selected preclinical studies addressing the influence of different dietary patterns and weight-loss strategies on adipose tissue renin–angiotensin system modulation.

Reference	Sample	Experimental Design	Observations on RAS
Gupte et al., 2008 [27]	Male C57BL/6 mice (8-week-old)	Mice fed with a LFD or a HFD (10 or 60% of energy as fat) for 1 week or 4 months.	↑ circulating levels of angiotensin peptides I, II and IV were found after 4 months of HFD feeding. ↑ gene expression of *angiotensinogen* (epididymal AT) and *Ace2* (epididymal and subcutaneous AT) in HFD fed mice after 1 week or 4 months. ↑ ACE2 protein expression in epididymal AT after 1 week of HFD feeding. ↓ ACE2 protein expression in epididymal AT after 4 months of HFD feeding.
Coelho 2010 [36]	Male Wistar rats	Rats fed with a standard diet for 1 months. A group of rats received sucrose in drinking water (20% solution).	↑ plasma angiotensin I and II levels in sucrose fed animals. ↑ plasma renin and ACE enzyme activities in sucrose fed animals. ↑ angiotensin I, II and (1-7) levels in epididymal AT of sucrose fed rats. ↓ protein expression of ACE2 in epididymal AT of rats receiving sucrose. ↑ ACE protein expression and activity in epididymal AT of rats receiving sucrose. ↓ *Agt* gene expression in epididymal AT of rats receiving sucrose. ↑ AT_1_ and AT_2_ protein expression in epididymal AT of sucrose fed rats.
Gupte et al., 2012 [33]	Male and female C57BL/6 mice (8-week-old)	Mice fed with a LFD or a HFD (10 or 60% of energy as fat) for 4 months.	↑ plasma angiotensin II levels in male mice fed the HFD compared to LFD-fed counterparts. ↓ plasma angiotensin (1-7) levels in LFD fed females compared to HFD fed males. ↓ plasma angiotensin (1-7) levels in HFD fed males compared to LFD fed males. ↑ plasma angiotensin (1-7) levels in HFD fed females compared to LFD fed males. ↑ AT ACE2 activity in HFD-fed males and females compared with LFD fed males.
Pinho 2013 [34]	Male FVB/N mice (8-week-old)	Mice fed with a STD, an AIN-93M, a HG, a HP or a HL diet for 2 months.	↑ *Ace* gene expression in the epididymal AT of mice fed the HL diet compared to the groups fed with the STD and AIN-93M diets. ↓ *Ace2* gene expression in epididymal AT of mice fed the HL diet compared to the animals fed the AIN-93M diet.
Zhang et al., 2014 [35]	Male Sprague-Dawley rats (8-week-old)	Rats fed with a STD or HFD (mainly lard) for 6 months	↑ *Ace2* gene and protein expressions in epididymal AT in HFD-fed rats.
De Almeida Pinheiro 2017 [37]	Male Swiss mice (4-week-old)	Mice fed with a STD or a HFD (61% of energy as fat) for 2 months. Animals fed with the two diets had free access to food or were submitted to 20, 40 or 60% of food restriction.	↑ *At1* gene expression in ad libitum HFD-fed mice compared to STD-fed controls. ↓ *Agt* and *Ace* gene expression in STD-fed animals submitted to energy restriction compared to ad libitum STD-fed counterparts. ↓ *Ace* gene expression in HFD-fed animals submitted to energy restriction compared to ad libitum HFD-fed counterparts.
Oliveira Andrade et al., 2014 [38]	Male FVB/N mice (4-week-old)	Mice fed with a STD or a HFD (11 or 61% of energy as fat) for 2 months. An additional group of animals fed with the HFD received 100 μg/kg bw/day of angiotensin (1-7).	↓ *Ace* gene expression in epididymal AT of mice fed the HFD compared to mice fed the STD diet. ↓ *Ace* and *Agt,* and ↑ *Ace2* gene expression in the epididymal AT of mice fed the HFD and treated with angiotensin (1-7), compared to the non-treated animals.
Crespo et al., 2017 [39]	Male Wistar rats (8-week-old)	Rats fed with a STD or a HFD (8 and 61% of energy as fat, respectively) for 2 months. Animals on each diet underwent L or SG. After surgical intervention, animals were fed the same diets for 1 additional month.	↓ Gene expression of Agt in periepididymal AT in STD and HFD fed rats with SG compared to animals with L fed the STD and HFD. ↓ Ace gene expression in periepididymal AT in STD- and HFD-fed rats with SG compared to animals with L fed the HFD. ↑ Ace2 gene expression in periepididymal AT in HFD fed rats with SG compared to animals with L fed standard diet.

#### 2.1.2. Energy Restriction

De Almeida Pinheiro et al. [37] studied the RAS in the adipose tissue of mice under different dietary patterns. For that purpose, male Swiss mice were distributed into eight experimental groups fed as follows: standard diet (66% carbohydrate, 23% protein and 11% fat, 3.95 kcal per g of diet) ad libitum; standard diet with a 20% of energy restriction; standard diet with a 40% of energy restriction; standard diet with a 60% of energy restriction; HFD (24% carbohydrate, 15% protein and 61% of fat, 5.28 kcal per g of diet) ad libitum; HFD with a 20% of energy restriction; HFD with a 40% of energy restriction; and HFD with a 60% of energy restriction. The length of the experimental period was two months. Thus, the authors not only studied the effects of HFD feeding on RAS, but also those of different degrees of energy restriction. At the end of the experimental period, as expected, body weight and epididymal, retroperitoneal and mesenteric adipose tissue weights, as well as adipocyte surface area, were reduced in mice under caloric restriction when compared to their respective controls. When groups fed ad libitum with the standard diet or the HFD were compared, the previously mentioned parameters were higher in the group fed with the HFD.

The expression of different genes involved in the RAS system and related to inflammation were measured in the epidydimal adipose tissue. When standard diet and HFD fed control groups were compared, *Agt*, *Ace* and *Ace2* levels did not differ between them. By contrast, gene expression of *At1* was higher in mice fed with the obesogenic diet than in mice fed with the standard diet. With regard to the effect of energy restriction, *Ace2* gene expression remained unchanged in mice fed with the restricted standard diets. Furthermore, *Agt* and *Ace* mRNA levels were reduced, with the exception of the highest restriction (60%) that did not modify their levels. In mice fed with the obesogenic diet, *Ace* mRNA levels decreased when the amount of food was limited, whereas *Agt* levels only decreased with the restriction of 40%. No differences were observed among groups in *Ace2* or *At1* mRNA levels.

With reference to inflammation, gene expression of *interleukin 6* (*Il-6*) and *tumor necrosis factor alpha* (*Tnfα*) were higher in mice fed with the obesogenic diet than in mice fed with the standard diet. Regarding energy restriction, mice fed with the 40% restricted diet with a standard composition showed decreased mRNA levels of *both Il-6* and *Tnfα*. When mice were fed with a high fat diet, *Il-6* gene expression was reduced under the caloric restriction of 40% and *Tnfα* under caloric restriction of 20% and 40%. In summary, mild to moderate restriction (mainly 40% of food restriction) improved inflammatory status and RAS components more than a severe restriction did and, in general, a weight reduction could exert beneficial effects on RAS system. Regarding *Ace2* mRNA levels, it was neither modified by the diet composition nor by the caloric restriction or body composition.

#### 2.1.3. Non-Dietary Treatments Focused on Body Fat Reduction

**Figure 3:**
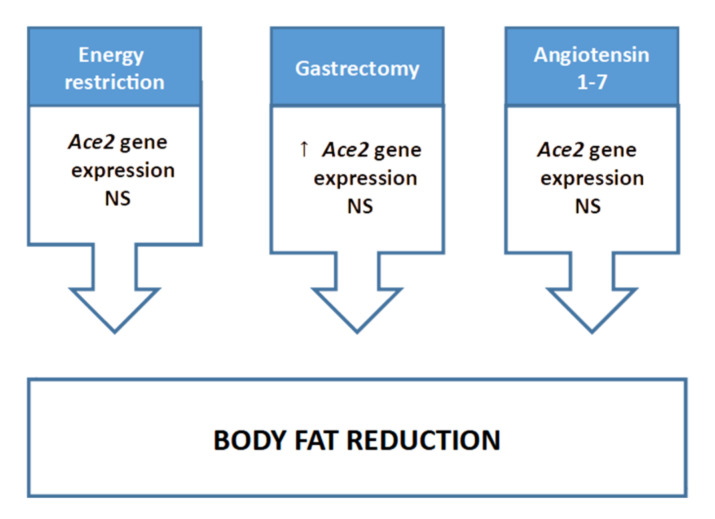
Effects of energy restriction and other non-dietary treatments devoted to reducing body fat on Ace2 gene expression in adipose tissue. NS: non-significant; ↑ increase.

In other studies, the effects of non-dietary treatments that reduce adipose tissue mass have been addressed (Figure 3). In this line, Oliveira-Andrade et al. [38] carried out a study to investigate the potential anti-obesity mechanisms of action of angiotensin (1-7). To do so, male FVB/N mice were fed with a standard or a HFD (11 and 61% of energy as fat) for two months. An additional group fed with the HFD received 100 μg/kg bw/day of angiotensin (1-7). The authors did not report any changes in food intake or body weight among the three groups. Epididymal, retroperitoneal and mesenteric adipose tissue weight was higher in mice fed with the HFD. By contrast, significant reductions in the three depots were found in the group fed with the HFD and treated with angiotensin (1-7) when compared with the non-treated animals fed with the same diet. Moreover, serum biochemistry analysis revealed that angiotensin (1-7) administration significantly reduced the increase in serum triglyceride, total cholesterol, glucose and insulin levels induced by the HFD, as well as improved glucose tolerance and insulin sensitivity tests.

When gene expression of different RAS components was studied in the epididymal adipose tissue, the authors found that whereas *Ace* gene expression increased under the high-fat feeding, *Ace2* and *Agt* gene expression remained unchanged when compared to mice fed with the standard diet. Angiotensin (1-7) administration significantly reduced *Ace* and *Agt* mRNA levels when compared to the non-treated HFD fed mice. In addition, a significant boost in *Ace2* gene expression was appreciated in these animals too. The authors also studied the protein expression of glucose metabolism markers in epididymal adipose tissue. No differences in protein expression of phosphorylated AMP-activated protein kinase (pAMPK), forkhead/wingedhelix O 1 (FOXO1) and glucose transporter 4 (GLUT4) were observed among animals fed with the standard diet or the high fat diet. Peroxisome proliferator-activated receptor γ (PPARγ) protein expression was higher in mice fed with the obesogenic diet. It has been observed that angiotensin (1-7) treatment induced a significant increase in the protein expression of pAMPK, FOXO1 and GLUT4, as well as a significant reduction of PPARγ protein expression when compared to mice fed with the same diet but not treated with angiotensin (1-7).

Altogether, the results revealed that, in this experimental model, high-fat feeding, which led to an increase in the size of epididymal adipose tissue, did not affect *Ace2* gene expression. Contrarily, oral angiotensin (1-7) administration, which reduced the size of this adipose depot and improved the impairments induced by the HFD feeding in glucose and lipid metabolism, did increase the expression of this gene.

In a recent study, Crespo et al. [39] explored the effect of sleeve gastrectomy (SG) on the metabolic profile and on the expression of inflammatory markers and RAS components in different tissues of rats featuring diet-induced obesity. Bearing this in mind, male Wistar rats were fed with a standard or a HFD (8 or 61% of energy as fat, respectively) for two months. After this experimental period, animals receiving both diets were divided in two groups that underwent a laparotomy or a SG. Following surgical intervention, animals were maintained in the same experimental diets for an additional month.

After surgery, and compared to the animals fed with the same diets that had undergone a laparotomy, a significant decrease in body weight and food intake (accompanied by reductions in body adiposity, adipocyte area and the weights of epididymal, mesenteric and retroperitoneal adipose tissues) were found among the animals that had been subjected to SG. Moreover, the pre-surgery impairment of glucose tolerance and insulin resistance produced by the HFD feeding were ameliorated in the animals that had SG. The authors also studied the gene expression of proinflammatory cytokines and RAS components in periepipidymal adipose tissue. The results revealed that the gene expression of *Tnfα* was significantly lower in rats fed with both diets and subjected to SG, when compared to the animals fed with the HFD that had undergone a laparotomy. Similar results were also reported for *Il-6* gene expression, although in this case the reduction reached significance only in the group fed with the HFD and subjected to SG. In the case of RAS components, a significant reduction in *Agt* gene expression was found among the animals fed with both diets and that had undergone SG, in comparison to their respective laparotomiced controls. In addition, and compared to the HFD fed laparotomiced animals, significant reductions in *Ace* mRNA levels were also appreciated in standard and HFD rats that had SG performed. By contrast, enhanced *Ace2* gene expression was appreciated in animals subjected to SG and fed with the HFD compared with their laparotomiced control counterparts and with animals that were fed with the standard diet and that had undergone SG.

The authors concluded that the body weight-loss and adipose tissue reductions produced by SG were accompanied by the improvement of the metabolic profile of the animals. Additionally, the authors also pointed out that the changes observed in adipose tissue inflammatory markers and RAS components may play an important role in the metabolic improvements derived from SG mediated weight-loss.

#### 2.1.4. Conclusions

**Figure 4:**
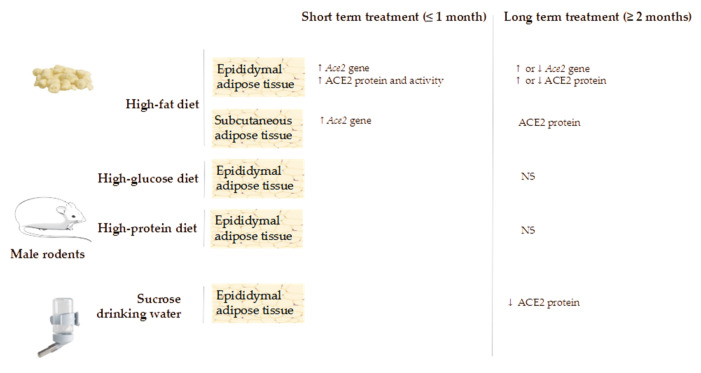
Effects of diet composition on ACE2 gene and protein expression in adipose tissue. NS: non-significant; ↑: increase; ↓: decrease.

To put all the aforementioned studies concisely (Figure 4), it can be stated that the composition of the diet, as well as the increase in adipose tissue mass, can effectively modulate the plasma levels and/or the adipose tissue expression/activity of RAS components, although a general consensus has not been reached. Regarding the ACE2 protein, has been related to SARS-CoV-2, Gupte et al. [27] and Olveira-Andrade et al. [38] observed an increase in *Ace2* gene expression in mice after four and two months of high-fat feeding, respectively. Contrarily, Pinho et al. [34] observed a decline after two months. Moreover, Alemida-Pinherio et al. [37] did not find significant differences after the two-month period. Zhang et al. [31] reported an increased *Ace2* gene expression in rats following a six-month period of high-fat feeding. The discrepancy among these results remains unclear. The fact that different species were used does not seem to justify such heterogeneous conclusions since Gupte et al. [27] and Olveira-Andrade et al. [33] observed the same results in mice than Zhang et al. [35] had observed in rats. Furthermore, the two-month experimental period used in the trials does not support such heterogeneity neither because the studies conducted by Oliveira et al. [38], Almeida et al. [37], and Pinho et al. [34] yielded divergent results too. Lastly, the anatomic location of the adipose tissue does not seem a plausible explanation for the conflicting findings since all the studies were carried out using epididymal adipose tissue. Dietary patterns rich in carbohydrates have barely been analysed. Coelho et al. [36] did not measure *Ace2* gene expression in rats, but they observed a decrease in ACE2 protein expression in rodents which received sucrose diluted in drinking water (20% solution). By contrast, Pinho et al. [34] did not find significant differences after two months of high-glucose feeding.

In addition to the effect of diet composition, other dietary interventions such as energy restriction have been studied. The reported data did not show any modification in *Ace2* gene expression, either under standard feeding or under high-fat feeding. Nevertheless, it is important to point out that this data comes from an isolated study and thus further research is required to reach a solid conclusion. The influence on *Ace2* of other non-dietary treatments that lead to a reduction in adipose tissue weight such as administration of angiotensin (1-7) or gastrectomy have also been reported. These have shown an increase in *Ace2* gene expression in adipose tissue [38,39]. Although a correlation analysis between adipose tissue size and *Ace2* expression has not been carried out in the aforementioned studies, the reported data cannot be used to hypothesize about the potential relationship between these two parameters.

### 2.2. Preclinical Studies Carried Out in Genetically Modified Rodent Models

Genetically modified rodent models have been used to address the potential relationship among ACE2, obesity and diet. Patel et al. [40] fed wild-type (WT) and ACE2 knockout (ACE2KO) male C57BL/6 mice with a standard diet (10% energy from fat) or a HFD (45% energy from fat) from weaning to six months. In ACE2KO mice fed with the HFD, a subcutaneous pump was placed to deliver Angiotensin (1-7) or saline (control) for four weeks. At the end of the experimental period, the HFD feeding increased body weight, fat content and epicardial adipose tissue to a similar level in both WT and ACE2KO mice. However, no differences in these parameters were observed in mice fed with a standard diet. Angiotensin (1-7) administration completely reversed body weight gain, while partially reversed the increment on body fat content. Fasting glucose was significantly increased in mice fed with the obesogenic diet but to a higher level in the ACEKO group. Intraperitoneal glucose tolerance test (IPGTT) area was only increased in ACE2KO mice that had been fed with the HFD. No data regarding fasting plasma glucose levels was provided in mice receiving Angiotensin (1-7), but a reduction in IPGTT area was observed.

In epicardial adipose tissue, adipocyte area was increased in HFD fed mice, and this effect was reversed by Angiotensin (1-7) but without reaching the values found in mice fed with the standard diet. Inflammatory markers were measured in this same adipose tissue. Immunofluorescent staining showed an increase in CD11c+/F4/80+ and CD206+/F4/80+ macrophage phenotypes in mice fed the obesogenic diet. The increase of CD11c+/F4/80+ was greater in ACE2KO mice while in the case of CD206+/F4/80+ the increase was lower. In this regard, it should be pointed out that F4/80 is a cell-surface marker of macrophages, CD11c is a marker of pro-inflammatory phenotype of macrophages and CD206 a marker of anti-inflammatory phenotype of macrophages. Taking that into account, these results could be indicating that ACE2KO mice are more prone to diet induced inflammation. It bears mentioning that Angiotensin (1-7) administration restored the levels of both macrophage ratios. In addition, different markers of inflammation and macrophage infiltration were measured by RT-PCR. *Monocyte chemoattractant protein 1* (*Mcp1*) expression was higher in mice fed the HFD, being this increase even higher in ACE2KO mice. This effect was completely reversed by Angiotensin (1-7) administration. Not only macrophage infiltration, but also gene expression of pro-inflammatory cytokines *Il-1β* and *Il-6* was higher in mice fed the obesogenic diet being this effect again avoided by Angiotensin (1-7) administration. TNFα protein levels and gene expression, as well as *inducible nitric oxide synthase* (*iNos*), and anti-inflammatory *Il-10* gene expression were only measured in ACEKO mice. In this regard, their levels were higher in ACE2KO mice without Angiotensin (1-7) administration than when this compound was administered or when compared to the mice fed the standard diet. Summarizing, ACE2 can negatively regulate HFD induced epicardial adipose tissue inflammation and Angiotensin (1-7) administration improves the inflammation.

Inflammatory markers revealed that ACE2KO mice were more prone to diet-induced inflammation than WT mice. The alterations induced by the high-fat feeding on these biomarkers were reverted by Angiotensin (1-7) administration. In this study, the authors found a novel role of ACE2 in obesity, where ACE2 negatively regulated obesity-induced epicardial adipose tissue inflammation, cardiac insulin resistance, and alterations in cardiac metabolism. Angiotensin 1-7 treatment improved these shifts. Consequently, the authors concluded that enhancing ACE2 or Ang 1-7 action represented potential therapeutic options for obesity and its associated heart disease. The effect on epicardial adipose tissue inflammation could be of interest in SARS-CoV-2 infection.

More recently, Shoemaker et al. [41] studied the effect of ACE2 in adipocytes. To do so, a mouse model of ACE2 deficiency was generated in both male and female C57BL/6 mice. ACE2 deficient and non-deficient mice were fed with a HFD (60% kcal from fat) or LFD (10% kcal from fat) for 16 weeks. At the end of the study, the body weight was greater in males than in females (independently of the diet). Body weight was also found to be increased in animals fed with the HFD than in mice fed with the LFD, without differences between both genotypes. Females fed with the LFD had lower percentage of fat mass than their male counterparts did. By contrast, female mice fed with the HFD had a higher percentage of fat than males. In both females and males, the percentage of fat mass did not vary in ACE2 deficient mice when compared to non-deficient mice, regardless of the diet they had been fed with. In view of these results, the authors concluded that adipocyte ACE2 does not seem to be protective in the development of obesity in female or male mice. This conclusion is not in good accordance with that of the study carried out by Shoemaker et al. [41].

## 3. Influence of Obesity and Diet in Adipose Tissue ACE2 Levels. Evidence from Studies Conducted in Humans

After having analysed to which extent RAS components can be modulated by diet and treatments devoted to reducing body fat in animal models, the next step is to address this issue in humans. Although the data available in humans is scarce in comparison to that obtained in animal models, some authors have investigated this topic. It must be noted that, due to ethical reasons, and contrarily to what happens in animal studies, no study conducted in humans was carried out where participants received high-fat or unbalanced diets. Therefore, the influence of diet in adipose tissue ACE2 expression has been based on the analysis of the outcomes found after weight-loss interventions.

As in rodents, it has been reported that the expression of ACE2 in human adipose tissue is greater than that occurring in lung tissue [42]. Similarly, by using publicly available subcutaneous adipose tissue transcriptomics datasets (GSE), it has been demonstrated that ACE2 expression is also modulated by diet in humans [43]. In addition, according to recently published data, it seems that interventions devoted to inducing weight-loss in humans can also modulate ACE2 expression in white adipose tissue. Thus, an *Ace2* gene expression decrease has been observed in subcutaneous adipose tissue of overweight or obese subjects of both sexes (28–35 kg/m^2^ BMI) that underwent weight-loss (five weeks following low-calorie diets). Additionally, it was also observed that this change in *Ace2* expression was maintained in subjects whose body weight-loss was more stable [43]. Similar results were found in a study in which overweight or obese subjects (BMI ≥ 27 kg/m^2^) were first subjected to a multimodal 12-week weight-loss intervention (low energy diet, nutritional counselling, physical activity and psychological support), and then maintained for a 12-month weight-maintenance period (with or without nutritional counselling) [44]. In this study, pre-intervention data revealed that, compared with healthy obese subjects, subcutaneous adipose tissue *Ace2* gene expression was lower in insulin resistant subjects. The weight loss induced by the multimodal intervention (at least 8% of body weight) significantly decreased subcutaneous adipose tissue *Ace2* gene expression. Remarkably, the authors found that the post-intervention improvement in insulin sensitivity was less marked in those subjects featuring a stronger reduction in adipose tissue *Ace2* gene expression [44].

Besides dietary interventions, bariatric surgery, such as Roux-en-Y gastric bypass (RYGB), represents an additional effective intervention tool to reduce adipose tissue volume. Therefore, RYGB is currently considered as a potential strategy to reduce COVID-19 risk of infection, especially in patients suffering from severe obesity [45]. Indeed, analysis on publicly available transcriptomic datasets (GSE59034) of subcutaneous white adipose tissue (sWAT) biopsies from 16 women before and 2 years after RYGB revealed that ACE2 gene expression is down-regulated by RYGB in this tissue. It is worth noting that the *Ace2* gene expression in these patients resulted even lower than that found in subcutaneous adipose tissue of subjects that had never been obese [45].

Altogether, the results of the aforementioned studies show that a reduction in adipose tissue weight is paralleled by a decrease in Ace2 gene expression in this tissue. Nevertheless, there are also studies that have found no *Ace2* gene modulation in patients under different nutritional status. De Almeida Pinheiro et al. [37] conducted a study in which the gene expression of several inflammatory markers and RAS components were measured in adipose tissue of eutrophic, obese and malnourished subjects. The authors found that compared with eutrophic subjects, gene expression of *Il-6*, *Tnfα*, *Agt* and *Ace* in visceral adipose tissue were significantly increased in obese and malnourished participants. According to the results previously described, increased *Ace2* gene expression would be expected in obese subjects. However, no differences among the three groups were found.

Moreover, a recent meta-analysis that compared data of *Ace2* gene expression in subcutaneous adipose tissue of participants in three cohorts (TwinsUK, METSIM and FUSION) that differed in geographic, demographic and phenotypic characteristics, reported that *Ace2* gene expression was negatively associated with BMI [46]. Indeed, the authors not only found that adipose tissue *Ace2* gene expression was lower in obese subjects than in normal-weight subjects, but they also observed that this parameter was even lower in those subjects featuring type 2 diabetes. Interestingly, the authors focused their attention both on the amount of adipose tissue of the subjects and on the cell type present in it. In this regard, the presence of microvascular endothelial cells (MVEC) was related to a higher *Ace2* gene expression, despite the fact that this expression was decreased when a greater macrophage level was present in the adipose tissue [46].

Ultimately, the data reported to date does not allow to draw a clear conclusion concerning the relationship between adipose tissue size and *Ace2* gene expression in this tissue and thus to link this relationship with an increased risk of being infected by SARS-CoV2 virus and the poor prognosis of COVID-19 amongst obese subjects.

In addition to the effect that dietary and non-dietary interventions devoted to reducing adipose tissue size can have on *Ace2* gene expression, some authors have focused their attention on the potential role of seaweeds, since in Japan and South Korea, two countries where seaweeds are commonly included in the diet, the number of deaths per 100,000 inhabitants have been clearly lower than in other countries were seaweeds do not take part of the usual dietary patterns [47]. Tanama (2020) has reported that seaweeds components appear to counteract the dominance of the ACE/Ang II/ATR1 axis in patients with COVID-19. Thus, the peptides in edible seaweeds might function as dietary ACE inhibitors, leading to a protective effect against COVID-19 by reducing the degree of ACE/Ang II/ATR1 axis dominance [48].

## 4. Influence of Obesity and ACE2 in COVID-19. beyond ACE2 Expression

As already mentioned in this review, the current knowledge on the mechanisms involved in SARS-CoV-2 infection points towards obesity as a risk factor for a greater viral entry. This assumption is based on the adipose tissue ACE2 expression and the abundance of such tissue in obese individuals. Additionally, a recent study has revealed that *Ace2* gene expression is enhanced in lung epithelial cells of obese subjects [43]. Moreover, in the same study, increased gene expression of *Tmprss2*, a serine protease known to be used by SARS-CoV-2 to bind S protein, was also found in the same cell line in obese subjects. These findings led to investigations that sought tissues that express ACE2 in order to find potential targets for viral infection. However, much care is needed when considering tissues with higher ACE2 expression as the main players in viral infection. In this line, it must be noted that whereas ACE2 expression in pulmonary tissue is lower than in other tissues (such as adipose tissue), the viral load usually found in the lungs is high. Similar observations have also been made with regards to the gastrointestinal tract, where the highest viral loads have been found in the colon, whereas the highest ACE2 expression occurs in the small intestine. These observations led to the idea that it is also possible to cause a productive SARS-CoV-2 infection with fewer ACE2 molecules [49]. Indeed, some studies carried out years before the outbreak of the COVID-19 pandemic suggested that ACE2 overexpression may actually exert a beneficial effect on lung inflammation [50].

With regard to the role of obesity in COVID-19, there is an overall consensus in terms of the negative effects of this metabolic disease on the progression and outcomes of the viral infection [51]. Interestingly, it has been proposed that the excess of adipose tissue, characteristic of obesity, may play a dual role in COVID-19. Thus, the reduction in ACE2 that occurs once SARS-CoV-2 enters the cell, results in increased levels of circulating angiotensin II, producing a pro-inflammatory response that may enhance lung tissue inflammation. By contrast, the increased ACE2 expression that has been proposed to occur in adipose tissue of obese subjects [52,53,54] may also result in higher angiotensin (1-7) levels, which is known to exert anti-inflammatory and antioxidant effects, and thus might help alleviating SARS-CoV-2 infection induced damage [9]. Moreover, it has been proposed that in terms of a greater risk for a SARS-CoV-2 infection, the activation level of ACE2 is as important as its expression. In this regard, metabolic disorders characterised by inflammation (such as obesity) may result in greater ACE2 shedding from adipose tissue. Since in adulthood, adipose tissue expansion occurs through adipocyte hypertrophy, a process that is related to greater adipose tissue inflammation [55], it is plausible to suggest that the adipose tissue inflammation that occurs in obesity could also reduce ACE2 functionality in this tissue, and thus somehow protect the host from a SARS-CoV-2 infection [9].

In addition, it has also been observed that soluble ACE2 may contribute to COVID-19 susceptibility, although its significance remains uncertain. In this sense, subjects with obesity and patients with COVID-19 have higher serum ACE2 levels [56,57,58,59]. The increased soluble ACE2 levels in COVID-19 patients may result from the cellular lysis that occurs when a severe infection takes place. Increased plasma levels of this protein seem to be associated with a higher severity and worse outcome of the disease [60]. By contrast, in other tissues such as lung or heart this increase could exert protective effects against the severity of the infection in part due to its anti-inflammatory and anti-hypertensive effect [61]. Nevertheless, serum ACE2 proceed from different tissues and thus it is not feasible to know the extent to which adipose tissue, or other tissues, contribute to ACE2 release into blood.

Sex is another factor to take into account in COVID-19 infection and development equation [9]. For instance, the greater visceral adipose tissue accumulation found in the upper body that is common in men has been widely linked to adipose tissue inflammation and further metabolic impairments [55]. Thus, it has been hypothesized that this specific fat accumulation pattern may result in decreased adipose tissue surface ACE2 expression, which in turn could reduce the risk of a viral infection. Indeed, the higher circulating ACE2 levels that are usually found in men would corroborate this assumption [9]. Moreover, due to hormonal (oestrogens upregulate ACE2 expression) and genetic factors (ACE2 is located in the X chromosome), ACE2 expression is greater in women, which may account for lower COVID-19 death rates than in men [62]. Additionally, lifestyle factors (such as tobacco smoking), as well as immune system functioning may provide women with more protection against a viral infection [62,63].

## 5. Conclusions

Regarding the first aim of the present review article, to analyse the current evidence with reference to the potential modulation that diet and obesity may exert in adipose tissue ACE2 expression, scientific evidence clearly shows that diet composition, mainly the amount of fat, affects this parameter. Unfortunately, the results available to date have been obtained in animal models and further studies are urgently needed to determine whether the reported effects are also induced in human beings. On the other hand, in humans, energy restriction leads to a reduction in *Ace2* gene expression, but the effects of dietary patterns other than restricted ones (i.e., Mediterranean diet and Western diet) should also be addressed in order to better understand to which extent ACE2 can be modulated through diet and to target dietary strategies with the aim of positively improving SARS-CoV illness severity.

Concerning obesity, the data reported demonstrate that the severity of COVID-19, as well as the outcomes of the disease, are worse in obese subjects. The results regarding the potential implication of ACE2 expression and modulation in COVID-19, obtained in preclinical studies or in studies carried out in humans, demonstrate that the shifts in ACE2 expression do not always occur in the same direction, nor with the same intensity.

With regard to the potential implications of ACE2 expression and modulation in COVID-19, there is also much debate. The greater risk of infection that hypothetically may derive from enhanced ACE2 expression is not clear, since the functionality of the enzyme seems to be as important as its abundance. Thus, the greater affluence of ACE2 in adipose tissue of obese subjects may be counterbalanced by its lower activation. In addition, a protective role of ACE2 overexpression has also been suggested, mainly due to the potential increase in anti-inflammatory factors that it may produce. Indeed, the role of ACE2 is still too vague and poorly characterized, and consequently this topic requires further investigation since a great heterogeneity of results has been obtained within obese subjects, especially regarding whether further metabolic disturbances besides obesity are present or not. Nevertheless, its involvement in the action of the virus seems evident.

Finally, it is important to highlight that the number of studies carried out in humans are very scarce so far and the results obtained in animal models cannot be easily extrapolated to human beings. This represents a clear limitation of the present review and further reviews are needed in the near future, when more data will be available, to draw stronger conclusions.
